# Purified Mushroom Tyrosinase Induced Melanogenic Protein Expression in B16F10 Melanocytes: A Quantitative Densitometric Analysis

**DOI:** 10.2174/1874104501812010036

**Published:** 2018-02-28

**Authors:** Kamal U. Zaidi, Sharique A. Ali, Ayesha S. Ali

**Affiliations:** 1 Biotechnology Pharmacology Laboratory, Centre for Scientific Research & Development, People’s University Bhopal-462037, Bhopal, India; 2 Department of Zoology & Biotechnology, Saifia College of Science, Bhopal-462001, India

**Keywords:** Tyrosinase expression, Density analysis, Western blot, Melanocytes, Absorption, Melanogenic protein

## Abstract

**Background:**

Human skin exists in a wide range of different colors and gradations, ranging from white to brown to black. This is due to the presence of a chemically inert and stable pigment known as melanin, which is produced deep inside the skin but is displayed as a mosaic at the surface of the body.

**Methods & Materials:**

In mammalian melanocytes, melanosome is a highly specialized organelle where melanin is synthesized. Melanin synthesis is controlled by tyrosinase, the vital enzyme in melanogenic pathway. The present investigation is based on the effect of purified tyrosinase of *Agaricus bisporus* on B16F10 melanocytes for melanogenic protein expression.

**Results:**

After the treatment of purified tyrosinase B16F10 melanocytes did not show any cytotoxic effect. Melanin content in B16F10 melanocytes was increased by purified tyrosinase in a dose-dependent manner. Quantitative western blot analysis revealed that cellular tyrosinase intensity was enhanced after treatment with purified tyrosinase for 48 hours, where the band intensity had a steady increase in the absorption of purified tyrosinase in B16F10 cells. The density analysis described increased absorption for 2 to 5 bands as 2.7, 3.7, 6.7 and 8.6% respectively. The bands in the comparative analysis of western blot were between the Rf value range (0.40-0.57) with maximum absorption of 3000 intensity curve at 32μg/mL, rather than higher concentration 64μg/mL, showing a decrease in the absorption.

**Conclusion:**

It is presumed that purified tyrosinase can be used as contestants for the treatment of vitiligous skin conditions.

## 1 INTRODUCTION

Defect in melanocytes or its functions results in pigmentary disorder leading to enhanced, reduced or complete loss of skin pigmentation. Individuals suffering from any of the hypo/depigmentary disorders, particularly disfiguring vitiligo are susceptible to the environmental assaults and cosmetic psychological stress. Thus, upregulating melanocyte activity in terms of growth and pigment synthesis in such condition is important. Reduced melanin is due to decrease / absence of melanocytes, leading to hypopigmentation disorders; hence development of melanogenic agents for photoprotection and hypopigmentation disorders is priority area in research [1]. In the era of modern medicine which is undergoing rapid change where genomic information is being accumulated, the data on vitiligo has not been appropriately archived or systemized for the disease analysis [2, 3].

Among several signaling pathways for enhancing melanin synthesis, the cyclic adenosine monophosphate (cAMP) pathway is a well-known signaling cascade and the most important pathway for regulating melanin synthesis [4]. cAMP-elevating agents; forskolin [5], IBMX [6], α -MSH [7], glycyrrhizin [8] can activate melanin synthesis by increasing cAMP levels. cAMP activates the Protein Kinase A (PKA) pathway. This leads to the phosphorylation of cAMP response binding protein (CREB) transcription factor that binds to cAMP Response Elements (CRE) on the MITF promoter and induces MITF expression. MITF then positively regulates the expression of tyrosinase, TRP-1, and TRP-2 involved in melanogenesis [9].

In melanocytes and melanoma cells, melanogenesis is controlled* via *a cascade of enzymatic reactions synchronized at the intensity of tyrosinase. The enzyme synthesizes dopaquinone from tyrosine, which is the rate limiting step of melanogenesis. Melanin synthesis is stimulated by many effectors, including 1-oleyl-glycerol [8, 10]. As confirmed, it becomes evident that extract from *Agaricus bisporus* has been used traditionally as well as medicinally in various ailments such as anti-tumor, immunomodulatory, hypocholesterolaemic, anti inflammatory, anti microbial and antiviral activities [11]. Despite this, to the best of our knowledge, there are no studies indicating extract of mushroom as melanogenic agent except for the work of Zehtab *et al* [12] who reported that mushroom tyrosinase prevented experimental autoimmune vitiligo. Suppression of clinical and histological disease was observed when animal received mushroom tyrosinase but exact mechanism is still unknown so an attempt is made to explore the detailed mechanism of mushroom tyrosinase on B16F10 melanocytes. The present study was under taken keeping the view of above lacunae in literature and for the first time B16F10 melanocytes model has been studied in detailed to mechanism of induced melanogenesis by purified tyrosinase of *Agaricus bisporus*. The findings of the study have provided vital information on these aspects and differentiating results have been obtained. It may be mentioned here that this is the first report of its kind where purified mushroom tyrosinase has been found to cause skin darkening* via *melanin displacement within the B16F10 melanocytes. The purified mushroom tyrosinase can be used as competent for the cure of hypopigmentation.

## 2 METHODS

For the present study, the compound, mushroom tyrosinase (lyophilized powder ≥1000 unit/mg solid), was purchased from Sigma-aldrich St. Louis, Missouri, United States. Goat anti-murine tyrosinase IgG antibody and Alexa Flour ® 594 dunkey anti-goat IgG (H+L) (2mg /mL) was purchased from life technologies North America, United States. Dulbecco’s Modified Eagle Medium (AT006A-5L) Fetal bovine serum (RM10432-100ML), Antibiotic Antimycotic Solution 100X (A002-20ML), Trypsin-EDTA solution1X (TCL042-5×100ML), 4', 6-diamidino-2-phenylindole (DAPI) (TC229-5MG), Phosphate buffered saline (RM7385-1PK) and Trypan blue, Certified (RM263-5G) were purchased from HiMedia Laboratories Pvt.Ltd. Mumbai.

### 2.1 Preparation of Tyrosinase

In the previous study, tyrosinase from *Agaricus bisporus* was purified by ammonium sulphate precipitation, dialysis followed by gel filtration chromatography on Sephadex G-100, and DEAE-Cellulose ion exchange chromatography [13].

### 2.2 Preparation of Melanocyte Culture

The melanocyte cell line B16F10 was procured from National Center for Cell Science, Pune and was cultured in Dulbecco’s Modified Eagle’s Medium (DMEM) containing 10% heat inactivated Fetal Bovine Serum (FBS), 1.5 g/L NaHCO3, 2mM L-glutamine, 10,000 units penicillin, 10 μg/mL streptomycin, and 25 μg/mL amphotericine B and incubated at 37*˚* C with 5% CO_2_ in a humidified atmosphere. To inhibit the bacterial contamination 2% Benzalkonium chloride was kept in incubator. The cells were subcultured in a ratio of 1:3 on every third day. For cell expansion and experiments with isolated cells, the B16F10 cells were detached with 1X Trypsin- (0.25% Trypsin and 0.1% EDTA in Hank’s balanced salt solution). After 3-4 passages, the cells were discarded and when necessary the cells preserved in liquid nitrogen were used as fresh culture.

### 2.3 Cell Viability Assay

When 70% confluency of B16F10 melanocytes was attained, trypsinization and seeding were done in 96 well microtitre plates at a density of 10^4^ cells/well in DMEM media supplemented with 5% FBS and 10,000 units penicillin, 10 μg/mL streptomycin, and 25 μg/mL amphotericine B antibiotic solutions. After overnight incubation, media of each well was replaced and the cells were treated with desired stimulants to perform MTT assay [14], to examine cytotoxic effect of extracted tyrosinase of *Agaricus bisporus *along with standard control tyrosinase (Sigma) in B16F10 cells over the concentration range of 1 to 64 μg/mL at different incubation periods of 24, 48 and 72 hr respectively. At the completion of incubation stimulant-containing media were discarded and fresh DMEM media containing 1 mg/mL of MTT was added to each well and incubated at 37 °C for 4 h. The solution was replaced with 0.04 N HCl–isopropyl alcohol solution and further incubated at room temperature for 30 min. Harvested solution was centrifuged at 11337 _× _g for 5 min and absorbance of supernatant was measured at 570 nm using micro plate ELISA reader.

### 2.4 Melanin Assay of B16 F10 Melanocytes

Melanin content was determined by the method of Tsuboi *et al* [15] with minor modifications. 1×10^5^ cells /well were seeded in 24 well tissue culture plates. After overnight incubation, the purified tyrosinase of A.* bisporus *along with standard control tyrosinase (Sigma) were added in different concentration 1 to 64 μg/mL for 24, 48 and 72 hours. After trypsinization an aliquot was divided into two parts. Five hundred microlitres was used for cell counting and the rest was centrifuged at 1677 _× _g for 5 min and lysed with 200 μL of 1N NaOH stirring at 80°C for 1h. Melanin content was measured at 405 nm using an ELISA reader

### 2.5 Determining Mechanisms of Tyrosinase Action by using Western Blotting

To determine the quantity and quality of tyrosinase expressed in the treated B16F10 melanocytes, Western blot analysis using the method of Nalv *et al*. [16]. B16F10 cells (1×10^5^ cells) were seeded in 24-well tissue culture plates in 1mL of DMEM media supplemented with 10% heat inactivated FBS and 10,000 units penicillin, 10 μg/mL streptomycin, and 25 μg/mL amphotericine B antibiotic solutions. Cells were treated with different concentrations of extracted tyrosinase of *A. bisporus* along with standard control tyrosinase (Sigma) range of 1, 2, 4, 8, 16, 32, and 64 μg/mL for 72 h. On incubation, the cells were trypsinized and washed twice with PBS buffer. The cells were lysed using ice cold lysis buffer **(**0.1M Tris-HCl buffer, 1% Nonidet P-40, 0.01% SDS, 100µM phenyl methyl sulfonyl fluoride and 1µg/mL Aprotinin) and centrifuged at 13000 rpm for 10 min at 4 C. Quantification of protein content in supernatant was done using method of Lowry *et al*. [10], 100 μg of total protein from each cell extract was electrophoresed in 12% SDS-PAGE, in mini gel electrophoresis system (GeNei). After electrophoresis, gels were kept in 1X transfer buffer (25mM Tris, 190mM Glycine, 20% Methanol and 0.1% SDS) at 4°C for 30 min and then blotted on polyvinylidene difluoride (PVDF) membranes (HIMEDIA) soaked with 1X transfer buffer, using a Transblot system (GeNei) at 15 volts for 25 to 30 min. The membranes were washed with 1X TBST pH 7.2 (137M Sodium Chloride, 20mM Tris, and 0.1% Tween 20) and blocked with blocking buffer (5% Skim milk, 137M Sodium Chloride, 20mM Tris, and 0.1% Tween 20**)** overnight at 4°C. Following blocking, the membranes were washed thrice in 1X TBST for 5 min each at room temperature. Firstly the membranes were washed and then treated with primary antibody buffer of goat anti-murine tyrosinase IgG (1:100 dilution, life technologies) with gentle agitation at 4˚C for 1h. After incubation with primary antibody, the membranes were washed thrice in 1X TBST for 5 min each at room temperature with gentle shaking. After extensive washing with secondary buffer of donkey anti-goat IgG conjugated with horseradish peroxides (1:2000 dilution, life technologies). The proteins were visualized using Gel documentation analyzer (BIORAD) and densitometry was performed using Quantity One software.

## 3 STATISTICAL ANALYSIS

The data is presented as mean ± SEM (standard error of the mean), where *n* represents the number of dose concentrations (treated) used for a particular experiment. Comparisons were made between treated and control groups by using Student’s *t*-test. All data were analyzed using GraphPad Prism-5 software (UK). *P*<0.005 indicates statistically significant difference.

## 4 RESULTS AND DISCUSSION

In the previous study tyrosinase of *A*. *bisporus* was purified, 16.36-fold to give 26.6% yield on total activity in the crude extract and final specificactivity of 52.19 U/mg with molecular weight of 95 kDa. The purified tyrosinase was optimized and the results revealed that the optimum values are pH 7.0 and temperature 35°C. The highest activity was reported towards its natural substrate, L-DOPA, with an apparent Km value of 0.933 mM [13].

### 4.1 Cell Viability Assay

To examine the cell viability due to the treatment of purified mushroom tyrosinase MTT cytotoxicity assays were performed in B16F10 cells. A wide concentration range of purified mushroom tyrosinase (1-64 μ/ml) was preferred. It was observed that cell viability significantly increased in B16F10 culture system at a higher concentration. After 24h of treatment, all the concentration of purified mushroom tyrosinase (1-64 μg/ml) showed significant cell viability 103%, to 121% respectively as compared to control as 100% (0.382±0.5mg/1×10^5^cells). Maximum cell viability was observed at concentration of 64µg/ml 121.2% (0.462±0.5mg/1×10^5^cells) (*p*<0.005) whereas, tyrosinase (Sigma) possessed maximum proliferation of 126.3% (0.482±0.5mg/1×10^5^cells) (*p*<0.005) with respect to control as 100%. Moreover, extending the period of treatment from 24 to 48 h and 72h; increase in the dose of purified mushroom tyrosinase above 4 μg/ml no further enhancement in cellular proliferation was observed; rather growth stimulation started to decline gradually above this dose reaching 128.9% (*p*<0.005) as compared to control as 100% value at the considerable higher concentration of 64 μg/ml Fig. (**1**). These findings are in agreement with those of Mallick *et al*. [17], who reported the similar effects of Placental Total Lipid Fractions (PTLF) on B16F10 melanocytes viability. It had also been reported by Lee *et al*., 2005 [8] that the serial concentration of glycyrrhizin 0.2, 0.5 and 1.0 mM significantly increased the cell’s viability in B16 melanoma cells but significantly decreased when at higher concentration. The present findings are in full agreement with those of Yoon *et al*. [18] who reported the effects of isopanduratin A on cell viability and percentages of viable melan-cells.

### 4.2 Melanin Assay of B16 F10 Melanocytes

**Fig. (1):**
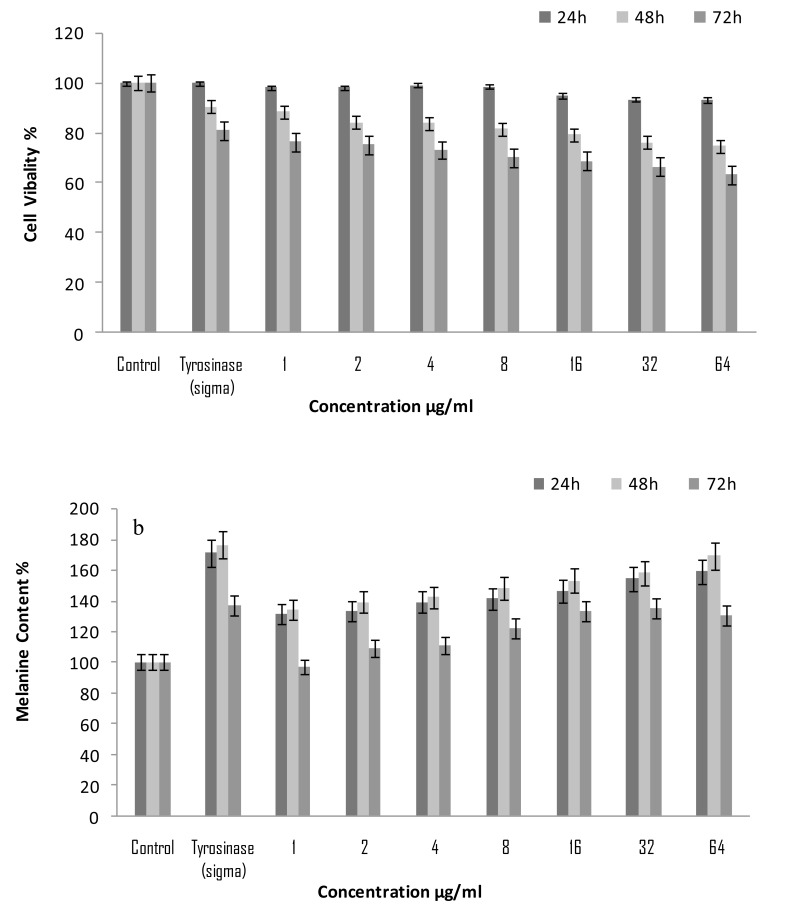
Purified mushroom tyrosinase induced pigmentation without affecting cell viability. Cell viability (a). Melanin synthesis in B16F10 melanocytes (b).Data represented as mean ± SE from triplicate experiments (*p*<0.005).

The pigment inducing ability of purified mushroom tyrosinase of *A. bisporuss* was estimated for melanin content in B16F10 melanocytes. When the extent of melanogenesis was examined in B16F10 cells after 24h of treatment with varying concentration of purified mushroom tyrosinase of *A. bisporus* (1-64 μg/mL); it was found to enhance melanin production in dose dependent manner within the concentration. It was observed that the concentration of 64μg/mL of mushroom tyrosinase of *A. bisporus *stimulated melanin synthesis maximally to 159% (0.656±0.5mg/1×10^5^ cells) (*p*<0.05) and minimum was 131% (0.541±0.5mg/1×10^5^ cells) (*p*<0.05) at concentration of 1 μg/mL. Moreover, tyrosinase (Sigma) was found to have more potent melanogenic activity (0.708±0.5mg/1×10^5^ cells) 171% (*p*<0.05) with respect to untreated control 100% (0.412±0.5mg/1×10^5^ cells) (Fig. **1b**). Whereas, extending the period of treatment from 24 to 48 h further enhancement of melanin production were observed in all the concentration of purified mushroom tyrosinase. Maximum melanin production was observed at 64 µg/mL 169% (0.697±0.5mg/1×10^5^ cells) (*p*<0.05) and standard control tyrosinase (Sigma) 176% (0.726±0.5mg/1×10^5^ cells) (*p*<0.05) (Fig. **1b**). After the treatment of 72h, melanin production in B16F10 cells was decreased in all the concentration. The present findings suggest that purified tyrosinase induced melanin production in the B16F10 melanocytes significantly, these are well supported by earlier classic work of Raman*****et al*. [19] who reported that aqueous extracts of *Angelica sinensis* root, a herb commonly used in the treatment of vitiligo in Traditional Chinese Medicine, was very active on mouse melanocytes. Similar data have been reported by Smith *et al*. [20] where Tomato Extract (TE) containing lycopene and Palm fruit Extract (PE) rich in carotenoids was used on the growth and pigmentation of melanocytes. Moreira *et al*. [21] who reported melanogenic activity of hydro alcoholic extracts from the leaves and flowers of *P. venusta* on murine B16F10 melanoma cells. Both extracts increased the melanin content in a concentration dependent manner after 4 days of incubation on melanoma cells.

### 4.3 Purified Tyrosinase of *A. bisporus* Specifically Accelerates the Absorption of Tyrosinase in B16F10 Melanocytes

#### 4.3.1 Densitometric Analysis of Western Blot of Treated B16F10 Melanocytes

**Fig. (2):**
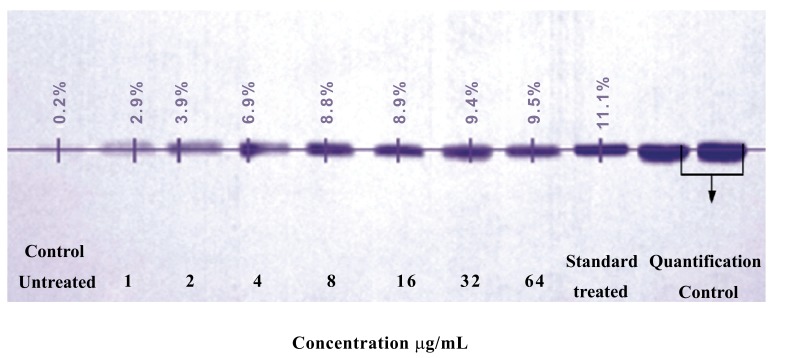
Desnsity analysis of Western Blot of treated B16F10 melanocytes. The inhibited cells (band 1) showed 0.2% band density revealing a trace amount of tyrosinase in the cell under study. The band intensity of 2-5 bands had a steady increase from 2.7, 3.7, 6.7 and 8.6% respectively showed the increased absorption with the increased quantity of treated tyrosinase. Rather (band 5-8) had an increase in the absorption quantity 8.7-9.3%; however the rate of increase is lower as compared to the bands 2-5. The average variations between the treatments were 1, 3, 1.9, 0.1, 0.5, and 0.1% respectively from the bands 2-7. The tyrosinase proteins were visualized by Quantity one software.

The average density of the band is the total intensity of the rows pixels which was used to generate the profile of a band, divided by the number of rows. The density of the band is directly proportional to the absorption of tyrosinase. The treated cell showed an increase in the absorption of tyrosinase which was indicated by the increased widthness of the bands of different treatment from 1-64 µg/mL concentration of purified tyrosinase for 48h incubation. The *A. bisporus* tyrosinase treated cells had an increasing absorption evident from the increase in the average density of the bands of tyrosinase formed by the different concentration (1 to 64µg/mL) incubated for 48 h incubation. The untreated cells (band 1) showed 0.2% band density. The band intensity of 2-5 bands had a steady increase from 2.7, 3.7, 6.7 and 8.6% respectively showed the increased absorption with the increased quantity of treated tyrosinase. Rather (band 5-8) had an increase in the absorption quantity 8.7-9.3%; however the rate of increase is lower as compared to the bands 2-5. The average variations between the treatments were 1, 3, 1.9, 0.1, 0.5, and 0.1% respectively from the bands 2-7 (Fig. **2**). The average rate of variation between the treatments got reduced after the treatment with 8μg/mL with a steady decrease to 0.1% difference between 8-16 μg/mL. It was observed that there were absorption of tyrosinase into the cell but was only 0.3% as average value. The cell treated with standard tyrosinase (sigma) of 64μg/mL showed an average intensity of (10.9%) which was higher than that of 64μg/mL (9.3%) of tyrosinase isolated from *A. bisporus.* However, the absorption ability of tyrosinase at 8µg/mL was (78.8%) in contrast to the standard control tyrosinase (100%) (Fig. **2**). The present findings of purified tyrosinase of *A. bisporus* inducing tyrosinase expression significantly in B16F10 melanocytes are in corroboration with the extensive work of Mallick *et al*. [17], where the effect of Placental Total Lipid Fractions (PTLF) was studied on B16F10 melanocytes. Taking the control as one fold, the increase in tyrosinase protein was found 3.06-fold in PP treated cells while treatment with PPPF showed 2.13-fold enhancement in tyrosinase protein expression by western blot analysis. The present findings showed that purified tyrosinase of *A. bisporus* induced tyrosinase expression in the B16F10 melanocytes are well supported with the work of Jeon *et al*. [22] who reported the effect of the main ingredients of lotus flower essential oil on tyrosinase expression. Tyrosinase expression was assessed by Western blot the intensity of each band was quantified by densitometry and normalized versus β-actin. The present findings showed that purified tyrosinase of *A. bisporus* induced tyrosinase expression in the B16F10 melanocytes are in corroboration with the recent work of Moleephan *et al*. [23] who reported the effect of xanthoxylin on B16F10 melanoma cells. It was observed that xanthoxylin significantly increased tyrosinase expression in a dose dependent manner when were quantified by western blot.

#### 4.3.2 Qualitative and Quantification Analysis of Western Blot of Treated B16F10 Melanocytes

**Fig. (3):**
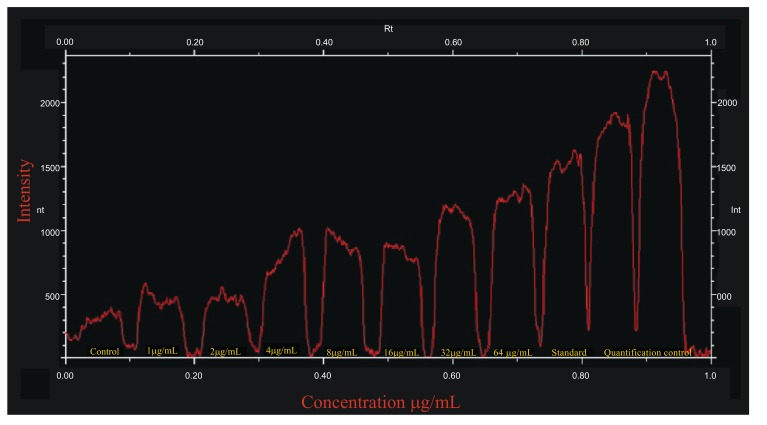
Qualitative analysis of Western Blot of treated B16F10 melanocytes. The untreated cell showed intensity of 200, indicated the cell tyrosinase that is over expressed in other treatment at different concentration. The plot shows a statically increase in the band intensity. At the concentration of 1, 2 and 4 μg/mL, an intensity of 450, 500 and 690 with a steady increase at 4 μg/mL was observed. On increasing concentration of purified tyrosinase of *A. bisporus* from 8 and 16 μg/mL treatment had the similar intensity value of 800. At highest concentration of 32 and 64 µg/mL of purified tyrosinase of *A. bisporus*, an increase of band intensity was observed with 1100 and 1200 which are fare lesser then that of standard control (Sigma) at 64μg/mL. The data obtained by using Quantity One software.

The bands are quantified by the average intensity value of each calculated band pixels. The number of pixel in a row of bands was determined and altogether, these results are the intensity profile for the band. The band quantity can then be quantified from the area under the Gaussian curve. The band intensity was plotted against Rf value of the bands to compare difference of absorption of tyrosinase enzyme of *A. bisporus* absorbed by the B16F10 melanocytes treated for 48h. The average intensity values of the band pixels produced by the B16F10 melanocytes treated with *A. bisporus* tyrosinase are plotted (Fig. **3**). It was observed that the untreated cell had intensity of 200, indicated the cell tyrosinase that is over expressed in other treatment at different concentration. The observation showed higher tyrosinase concentration then that of the negative control, presenting the absorption of *A. bisporus* tyrosinase. The plot shows a statically increase in the band intensity. The cell with standard tyrosinase (Sigma) treatment at a concentration of 64μg/mL had an absorption intensity of 1500 which is a higher intensity than the 64μg/mL purified tyrosinase of *A. bisporus* (Fig. **3**). At the concentration of 1, 2 and 4 μg/mL, an intensity of 450, 500 and 690 with a steady increase at 4 μg/mL was observed. On increasing concentration of purified tyrosinase of *A. bisporus* from 8 and 16 μg/mL treatment had the similar intensity value of 800. At highest concentration of 32 and 64 µg/mL of purified tyrosinase of *A. bisporus*, an increase of band intensity was observed with 1100 and 1200 which are fare lesser then that of standard control (Sigma) at 64μg/mL. These observations prove that *A. bisporus* tyrosinase has poorer absorption ability as compared to the positive control tyrosinase (Fig. **3**). The data of the present findings regarding qualitative and quantitative analysis of Western Blot of *A. bisporus* absorbed by B16F10 melanocytes are very well supported by those of Singh *et al*. [24], who clearly indicated that the B16F10 cells when inhibited with protein kinase will be under suppression for tyrosinase production and can be analyzed using Western blot to measure the amount of enzyme in the cells based on the intensity of the produced bands. The present findings have clearly demonstrated that qualitative and quantitative analysis of Western Blot of *A. bisporus* as absorbed by B16F10 melanocytes are in contrast with the recent work of Singh *et al*. [25] who reported the different plant extracts examined for their effect on melanogenesis in human melanocytes using Western blotting with densitometric analysis.

#### 4.3.3 Comparative Analysis of Western Blot of Treated B16F10 Melanocytes

**Fig. (4):**
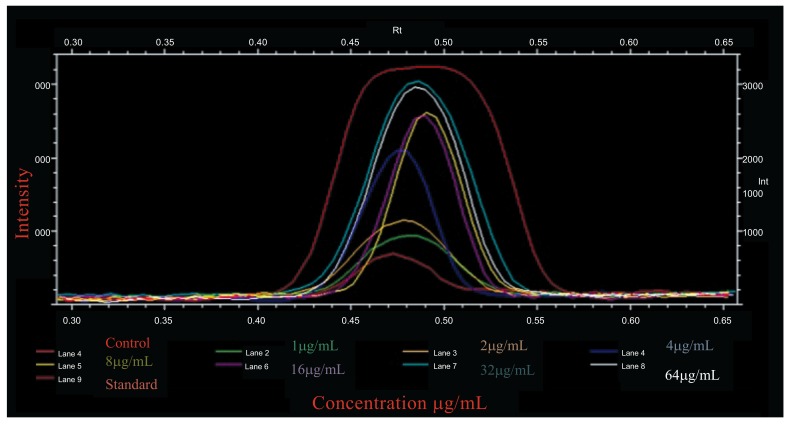
Comparative analysis of Western Blot of treated B16F10 melanocytes. The intensity profiles of all bands of absorption of tyrosinase of *A. bisporus* were compared on lanes graph range of intensity values. The bands were between the Rf value ranges 0.40-0.57 with maximum peak of 3200 intensity with standard tyrosinase (Sigma). The untreated cells had an intensity of 790 which was observed in all treatments with the increase of tyrosinase absorption for 1, 2, 4, 8, 16, 32 and 64 μg/mL as 950, 1100, 2000, 2590, 2600, 3000 and 2900 intensity respectively. A maximum absorption of 3000 intensity curve was shown by 32μg/mL, rather the higher concentration 64μg/mL showed a decrease in the absorption. The data obtained by using Quantity One software.

The compare lanes graph superimpose the intensity profiles of all band images. The X axis of the graph is the Rf value and the Y axis is the pixel intensity value at each point along the lane. Compare lanes maximize the range of intensity values included in the graph. The intensity profiles of all bands of absorption of tyrosinase of *A. bisporus* were compared on lanes graph range of intensity values. The bands were between the Rf value ranges 0.40-0.57 with maximum peak of 3200 intensity with standard tyrosinase (Sigma). Whereas, the untreated cells had an intensity of 790 which was observed in all treatments with the increase of tyrosinase absorption for 1, 2, 4, 8, 16, 32 and 64 μg/mL as 950, 1100, 2000, 2590, 2600, 3000 and 2900 intensity respectively. A maximum absorption of 3000 intensity curve was shown by 32μg/mL, rather the higher concentration 64μg/mL showed a decrease in the absorption (Fig. **4**). The absorption rate of standard tyrosinase at 64μg/mL is much higher than that of *A. bisporus* tyrosinase even at 32μg/mL. A decrease in absorption was absorbed to the 64μg/mL indicating a lesser absorption rate of the enzyme purified from *A. bisporus.* The data of the present findings showing comparative analysis of Western Blot of *A. bisporus* well absorbed by B16F10 melanocytes are in contradiction with those of Oh *et al*. [26] who reported that *Ficus deltoidea* extract exerted anti melanogenic activity by preventing tyrosinase activity *in vitro* and by suppressing tyrosinase gene expression in B16F1 melanoma cells. Villareal *et al*. [27] have demonstrated that the activation of MITF by *Argania spinosa* oil leads to the inhibition of the tyrosinase and dopachrome tautomerase expressions in B16 murine melanoma cells. Western blot analysis showed that the expression levels of Tyrosinase (TYR), Tyrosinase-Related Protein 1 (TRP1), and dopachrome tautomerase (DCT) proteins were decreased. In addition, there was an increase in the activation of MITF and ERK1/2. Choo *et al*. [28] have also reported the hypopigmenting effect of sesquiterpenes from *Inula britannica* in B16 melanoma cells. It was found that the Western blot analysis indicated that active compound inhibited melanogenesis by activating Extracellular signal-regulated Kinase (ERK) and Akt signaling and also inhibiting cAMP related binding protein.

In 2016 Zaidi *et al*. [29] have demonstrated morphoanatomic effects of purified tyrosinase to determine its skin-darkening potential using B16F10 melanocyte. After immunofluorescence microscopic observation, it was found purified tyrosinase increase cellular tyrosinase expression in doze dependent manner due to tyrosinase absorption in B16F10 melanocyte.

#### 4.3.4 Concentration Data Analysis of Western Blot of B16F10 Treated Melanocytes

A volume of a band is the intensity data inside of a band and it is the sum of the intensities of the pixels within the volume boundary and the pixel area (in mm^2). The volume is similar to band contours. To measure the amount of a particular band it is compare the intensity data inside the boundary with the data of other objects or a standard using the volume analysis report and volume regression curve. The volume concentration of each band were determined using the adjusted volume, mean intensity of the band pixels, area of the band, the minimum and the maximum intensity pixel in the band volume, the total intensity of all the pixels in the band volume divided by the area of the band volume and the width and height of the band volume in mm. The concentration of the bands of absorption of tyrosinase of *A. bisporus* were also increasing 92.5, 133.4, 286.7, 328.2, 343.6, 399.4 and 418.0 μg/μL for different concentration from 1-64 μg/mL treatment respectively. The untreated cell had the volume concentration of 41 μg/μL which was 2 fold lesser than the 1 μg/mL treated cell (Table 1), rather the positive standard tyrosinase (Sigma) showed a higher band concentration of 589.5 μg/μL with 64 μg/mL tyrosinase treatment.

The present findings confirmed that concentration data analysis of Western Blot of *A. bisporus* absorbed by B16F10 melanocytes. It can be correlated to the findings of Jeon *et al*. [22] which have explored lotus flower essential oil for tyrosinase expression in B16F10 melanocytes. Similarly, the present findings are in collaboration with those of the work of Tuerxuntayi *et al*. [30] who have reported that the Kaliziri extract upregulates tyrosinase, TRP-1, TRP-2 and MITF expression and the signaling pathway of melanin synthesis in murine B16 melanoma cells using western blotting. The absorption mechanism by which tyrosinase of *A. bisporus* stimulates melanin synthesis in B16F10 melanocytes remains to be identified. To achieve this aim, effect of mushroom tyrosinase was evaluated and characterized by western blot. Western blot indicated that the cells had absorbed the mushroom tyrosinase and the process of melanogenesis occurred within the cells. The study was also assessed for the rate of absorption of purified mushroom tyrosinase of *A. bisporus* and commercial standard tyrosinase (Sigma); it was observed that the standard tyrosinase possessed higher absorption might be due to its purity. Simultaneously, the present findings are also in full agreement with those of Zaidi *et al*. [31] who reported the effect of purified mushroom tyrosinase on melanin content and melanogenic protein expression. Using B16F10 melanocytes showed that the stimulation of melanogenesis by purified tyrosinase is due to increased tyrosinase absorption.

## CONCLUSION

Due to the lack of success in hypopigmentation, melanogenic protein expression in B16F10 melanocytes has been investigated using quantitative densitometric analysys. The present findings of the study have provided vital information on these aspects and differentiating results have been obtained. It may be mentioned here that this is the first report of its kind where purified mushroom tyrosinase has been found to cause skin darkening* via *melanin displacement within the B16F10 melanocytes. From the data of the present work, it is also concluded that tyrosinase is the key in the regulation of melanin production; the study conducted on the effect of purified tyrosinase absorption and its effects has shown that mushroom tyrosinase is an effective agent for melanogenesis and can serve as a potent melanogenic agent to cure hypopigmentation diseases.

## Figures and Tables

**Table 1:** Concentration data analysis of Western Blot of B16F10 melanocytes treated cells using Quantity One software.

**Index**	**Name**	**Adj. Vol.** **INT mm^2^**	**% Adj.Vol.**	**Concentration**	**Area** **mm^2^**	**Mean Value** **INT**	**Min. Value INT**	**Max. Value** **INT**	**Density** **INT/ mm^2^**	**Width** **mm**	**Height** **mm**
**1**	Std1	30082.01	38.73	1000.00	18.14	3279.32	2133.00	3782.00	327932.45	7.80	2.70
**2**	Std2	27242.00	35.08	750.00	17.66	3163.58	2003.00	3753.00	316358.20	8.10	2.50
**3**	Std3	20339.23	26.19	589.51	16.18	2878.05	1771.00	3742.00	287805.98	9.00	2.30
**4**	U1	6755.54	6.86	41.01	6.89	2601.48	1967.00	3069.00	260148.46	7.50	1.50
**5**	U2	8089.21	8.22	92.51	12.61	2262.49	1478.00	2808.00	226249.16	8.50	1.80
**6**	U3	9147.14	9.29	133.37	10.13	2523.97	2173.00	3040.00	252397.52	8.20	1.60
**7**	U4	13116.71	13.32	286.66	13.61	2584.75	1863.00	3651.00	258475.52	8.00	2.30
**8**	U5	14217.26	14.44	329.16	16.85	2464.75	1435.00	3587.00	246475.42	8.00	2.80
**9**	U6	14589.50	14.82	343.53	12.99	2744.13	1798.00	3561.00	274413.30	7.80	1.90
**10**	U7	16034.90	16.28	399.35	13.77	2785.48	1578.00	3545.00	278548.06	8.00	2.10
**11**	U8	16516.79	16.77	417.96	13.22	2870.37	2753.00	2990.00	287037.88	8.60	1.80

Intensity (INT)

## LIST OF ABBREVIATIONS

MITF: = microphthalmia-associated transcription factor
TYR: = tyrosinase
TRP1: = tyrosinase-related protein 1
TRP-2: = tyrosinase-related protein 2
DCT: = dopachrome tautomerase
ERK: = extracellular signal-regulated kinase
cAMP: = cyclic adenosine monophosphate
Akt: = protein kinase; *A. bisporus, Agaricus bisporus*
*A. bisporus*: *= Agaricus bisporus*
α-MSH: = α- melanocyte-stimulating hormones;
ACTH: = Adrenocorticotropic hormone; and
ET-1: = Endothelin-1;
PTLF: = placental total lipid fractions;
PP: = placental peptide
EDTA: = ethylenediaminetetraacetic acid
DMEM: = dulbecco’s modified eagle’s medium
FBS: = fetal bovine serum
DEAE: = diethylaminoethyl cellulose
DAPI: = diamidino-2-phenylindole.

## AVAILABILITY OF DATA AND MATERIALS

The datasets supporting the conclusions of this article are included within the article.

## AUTHOR DETAILS

^1^Biotechnology Pharmacology Laboratory, Centre for Scientific Research & Development, People’s University Bhopal-462037, India. ^2^ Department Biotechnology, Saifia College of Science, Bhopal-462001, India. 3Department of Zoology, Saifia College of Science, Bhopal-462001, India
